# Solvent extraction of Cu, Mo, V, and U from leach solutions of copper ore and flotation tailings

**DOI:** 10.1007/s10967-017-5383-y

**Published:** 2017-08-03

**Authors:** Tomasz Smolinski, Danuta Wawszczak, Andrzej Deptula, Wieslawa Lada, Tadeusz Olczak, Marcin Rogowski, Marta Pyszynska, Andrzej Grzegorz Chmielewski

**Affiliations:** Institute of Nuclear Chemistry and Technology (INCT), 03–195 Warsaw, Poland

**Keywords:** Copper flotation tailings, Extraction, Hydrometallurgy, Radiotracer

## Abstract

Flotation tailings from copper production are deposits of copper and other valuable metals, such as Mo, V and U. New hydrometallurgical technologies are more economical and open up new possibilities for metal recovery. This work presents results of the study on the extraction of copper by mixed extractant consisting *p*-toluidine dissolved in toluene. The possibility of simultaneous liquid–liquid extraction of molybdenum and vanadium was examined. D2EHPA solutions was used as extractant, and recovery of individual elements compared for the representative samples of ore and copper flotation tailings. Radiometric methods were applied for process optimization.

## Introduction

Hydrometallurgical processes are often coupled to pyrometallurgical ones (e.g., the electrochemical refining of metals obtained by pyrometallurgical process, or roasting and high-temperature reduction of hydroxides precipitated from solutions obtained by ore leaching) [1–4]. The main advantage of the hydrometallurgical process is that it allows processing of raw materials, for which the use of conventional methods is not economical or is troublesome [5–7]. Hydrometallurgical processes are used for recovery of many metals, including uranium, copper, cobalt, nickel, zinc, aluminium, titanium, gold and lanthanides. Moreover, they are used in the nuclear industry for the processing of spent nuclear fuel and the recovery of thorium, zirconium and hafnium [9–11].

Hydrometallurgical processes are multistep. Ore or concentrate is crushed and milled, and subjected to enrichment by physical processes and subsequent leaching. At this stage, metal compounds insoluble in water are converted into soluble salts suitable for other operations. The composition of the leach solution is chosen depending on the type of raw material subjected to leaching. These can be acids: H_2_SO_4_, HCl, HNO_3_; hydroxide: NaOH; chlorides: NH_4_Cl, FeCl_3_; or carbonates: Na_2_CO_3_, (NH_4_)_2_CO_3_ [12–16]. Recently, growing technological importance has been given to bacterial leaching, called bioleaching, using suitable strains of bacteria that act as biological catalysts (biocatalysts) [17]. The resulting aqueous solution contains ions of many metals, which have to be selectively isolated. For this purpose one of the following processes: extraction, membrane processes, ion exchange, cementation, crystallization, precipitation, can be used or other hybrid techniques applied.

The extraction of metal ions from aqueous solution is a prospective method for the concentration, separation and preparation of high purity metal. It opens up the possibility of using poor mineral resources, semi-finished products and industrial waste containing small quantities of the metals. For isolation and separation of ions, particularly multiple folded extractants are useful for process applications [18]. Moreover, radiotracer methods are a suitable tool for process investigation, since most of the elements involved may be activated in the nuclear reactor and their radioactive isotopes easily detected [10].

Today, around 25% of the world’s copper is recovered by solvent extraction, which is considered the lowest-cost production route for the production of high quality cathode material. In Chile, the world’s largest copper producer, solvent extraction process (SX) will soon be used to produce 50% of that country’s copper. Therefore, attention has to be drawn towards the development of suitable methods for leaching copper minerals as well as to developing and refining extraction methods [19].

KGHM Co. Poland was the eighth-largest copper producer and third-largest silver producer in the world in 2014 [20]. The primary technology used by the company is pyrometallurgy. Copper ore is crushed, ground and enriched by flotation and directed to the following stages of the pyrometallurgical process. During the preliminary stages of copper ore enrichment, significant amounts of flotation waste are generated. KGHM, since its inception, has been searching for a technology that is technically, economically and environmentally feasible for the economical usage of the waste from the flotation of copper ore [19].

The Institute of Nuclear Chemistry and Technology (INCT) has long experience in the application of different hydrometallurgical processes for metal recovery [3]. The INCT has been studying the possibility of the recovery of uranium and other elements produced as by-products of the preparation of copper concentrate from copper ore mined in Zagłębie Lubińskie in recent years [21–23].

## Materials and methods

### Reagents and apparatus

Toluene ACS, benzoic acid ACS, *p*-toluidine 99.6%, di-(2-ethylhexyl)phosphoric acid (D2EHPA) 97%, H_2_SO_4_ ≥ 97.5%, Na_2_CO_3_ ≥ 99.5% and MnO_2_ 99.9% were purchased from Merck. For preparation of the solid samples for analyses, analytical grade reagents: Na_2_O_2_, HNO_3_, Li_2_B_4_O_7_, LiBO_2_ were used. Distilled water was used in all experiments. The solvents were saturated with each other before use in order to prevent volume changes of the phases during extraction.

The concentration of Cu was analyzed and calculated using neutron activation analyses and confirmed by atomic absorption spectroscopy analyses (AAS Type Solar Series M-II-K manufactured by Thermo Electron Corporation). The activity measurements were performed using a Canberra gamma spectrometer with Ge detector. The content of U, Mo and V was chemically analysed by ICP-MS instrument (ELAN DRC II PerkinElmerTM) with a cross-flow nebuliser and a Scott double-pass spray chamber and Ni cones was used in the measurements. Standard solutions (1 mg mL^−1^) used in ICP-MS analyses were supplied by PerkinElmer. Raw materials were characterized also by thermogravimetric analysis (TGA) and differential thermal analysis (DTA) in a Hungarian MOM Derivatograph, for which the sample weight = 200 mg, heating rate = 10 °C/min, atmosphere = air, and reference material = Al_2_O_3_.

### Raw materials and leaching procedure

The aim of this study was to develop a method of recovery of various valuable metals from copper ores and tailings. The samples used in experiments were collected from the Polish Geological Institute—National Research Institute. The main components of the samples of ore from deposits in the Legnicko-Głogów District are: Chalcocite (Cu_2_S) and bornite (Cu_5_FeS_4_), easily leachable minerals predominate in the Polish ores, while content of chalcopyrite (CuFeS_2_) and covellite (CuS) is significantly lower [7, 24].

**Fig. 1:**
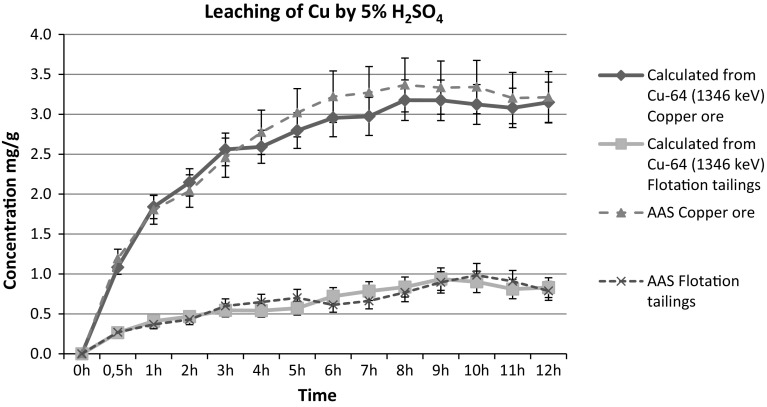
Results of leaching Cu from raw materials calculated from gamma spectrum

The samples of flotation tailings were taken from wastes collected from the landfill reservoir in Gilów. Samples of the raw material were ground to a powder and then dried at 110 °C for 2 h, then analyzed with the use of ICP-MS. The uncertainty of elemental determination by this technique was evaluated at 5–10% depending on the element: U- 5%; V- 8%; Mo- 10%, and due to the high inhomogeneity of the material. The optimum leaching conditions have been selected [8]. Samples were prepared in accordance with the following procedure: to 500 g of the powdered sample were added 250 mL of concentrated H_2_SO_4_ and 10 g of MnO_2_ as an oxidizing agent and the mixture was sintered at 150 °C for 8 h. Subsequently, leaching was performed by 1000 mL 5% H_2_SO_4_ for 12 h (optimally 10 h) at 25 °C and the solution filtered on a Büchner funnel. The processes were optimized using radionuclide ^64^Cu as a radiotracer. The samples of the raw material were activated in a research nuclear reactor (Maria Reactor in Świerk, Poland). Activation conditions were: neutron flux 10^14^ n cm^−2^ s^−1^, time 15 min. After 75 h of cooling the activity of the ^64^Cu was 21.5 MBq, T_1/2_ = 12.8 h. The concentration of Cu can be calculated according Eq. (1) and are shown in Fig. 1. These calculations were confirmed by AAS analyzes.

1 $${X} = \frac{{\frac{P}{\text{DCm}}}}{{\frac{{{P}_{0} }}{{{\text{DCm}}_{0} }}}}$$

*X* concentration of element, *P* counts number of radionuclide X, *P*_0_ counts number of radionuclide X standard sample, *D* a half-life correction for radionuclide, *C* factor responsible for the decay of the radionuclide at the time of measurement, *m* [g] mass of the sample, *m*_0_ [g] mass of the standard sample.

### Extraction procedure

The extraction process was performed using an extractant consisting of a mixture of benzoic acid (0.5 mol dm^−3^), an aromatic amine *p*-toluidine (0.25 mol dm^−3^), and an aromatic solvent, toluene, with a ratio of organic phase to aqueous phase of 1:1. Cu(II) ions were extracted two times with an aqueous solution of sulfate. The pH of the aqueous phase was regulated in the range 3.6–3.8 with sodium carbonate. After separation of the copper ions, other metal ions, such as nickel(II) and cobalt(II) may be extracted [25]. The process was realized by shaking in a mechanical shaker at 25 °C. Optimum equilibration time was determined for this system. In most cases distribution equilibrium was attained in less than 60 min and a shaking time of 60 min. The process of separation of ions was carried out until the degree of separation of metal ions is higher than 99%. In this work, we confined ourselves to the extraction of copper(II) from samples tested.

For recovery of V, Mo, U a diferent extractant was investigated. Experiments with D2EHPA in toluene were carried out. Concentrations of solvents used in the experiments were 0.3, 0.2, and 0.1 mol dm^−3^. Each of the three stages of extraction was carried out for 15 min with a ratio of organic phase to aqueous phase of 1:1. For the extraction, 15 mL of sample was taken. The time required for complete separation of the phases was 30 min. The organic phase was back-extracted with 5% Na_2_CO_3_ for 15 min. After thirty minutes phase separation time, allowed for next extraction step.

The distribution ratio (D) and extraction percentage efficiency (E) were calculated. The distribution ratio D is defined as:

2 $$D = \frac{{C_{M\,org} }}{{C_{M\,aq} }} = \frac{{C_{M\,total} - C_{M\,aq} }}{{C_{M\,aq} }} = \frac{{A_{org} V_{aq} }}{{A_{aq} V_{org} }}$$

where *C*_M org_ represents the concentration of copper into organic phase, *C*_M aq_ represents the concentration of copper into aqueous phase, *C*_M total_ represents the total concentration of copper. *D* values can be evaluated using activity of the radionuclide where *A* and *V* represent radioactivity and the volume of the solution, respectively.

In order to better characterise the efficiency of the extraction process, we introduced the concept of extraction percentage (% E), which is given by:

3 $$\% E = \frac{D*100\% }{{D + {\raise0.7ex\hbox{${V_{aq} }$} \!\mathord{\left/ {\vphantom {{V_{aq} } {V_{org} }}}\right.\kern-0pt} \!\lower0.7ex\hbox{${V_{org} }$}}}}.$$

## Results and discussion

### Raw material leaching

Thermogravimetric analyses (TG, DTA) of raw materials were carried out, under which specified temperature changes that occur during the calcinations were observed. The water content of the samples was low (below 5%). The thermal degradation pattern of ore sample presents a clearly visible exothermic peak at 470 °C, probably related to the decomposition of sulfides, carbonates, the burning of coal or other organic matter (about 10%). The stabilisation at the sample mass ratio at ca. 78% occurred at 850 °C. Loss of mass in the flotation tailings sample was less, and no exothermic effects were observed. Stabilization of sample mass, at approximately 90%, occurred at 850 °C.

The analysis of the test samples of starting materials of individual elements showed several metals in the samples and that copper was in majority. In this paper we focused on presenting the results of extraction of the following elements: Cu, Mo, V and U. Also noteworthy is the fact that other elements could be recovered as well as copper. The results of leaching experiments are shown in Table 1. Smiliar results of the research regarding starting material are contained in our earlier work [12]. In this study for determination of Cu concentration we used nuclear activation analyses instead AAS.

**Table 1:** Results of analysis of selected elements in copper ores and copper flotation wastes

	Cu ore	Metal concentration in leach liquor	Flotation tailings	Metal concentration in leach liquor
Cu (ppm)	45070 ± 3505	33802 ± 2704	13320 ± 1066	9979 ± 798
V (ppm)	1296 ± 103.7	698 ± 55.9	51 ± 4.1	22 ± 1.8
Mo (ppm)	346 ± 34.3	293 ± 29.3	25 ± 2.5	15 ± 1.6
U (ppm)	26 ± 1.3	14 ± 0.7	5,5 ± 0.3	2 ± 0.1

### Cu extraction

So far, for the isolation of Cu, in acid leach solutions, oximes were used [26–29]. The chemistry of oxime extraction of copper is relatively simple (Eq. 4).

4 $$2{\text{RH}}_{{({\text{org}})}} + {\text{Cu}}^{2 + } + {\text{SO}}_{4}^{2 - } \to {\text{R}}_{2} {\text{Cu}}_{{({\text{Org}})}} + 2{\text{H}}^{ + } + {\text{SO}}_{4}^{2 - } .$$

For metal extraction from ammoniacal solutions, beta diketones may be used. Adducted extractants, because of their complex composition, are difficult to obtain and analyses, and are further characterized by low extraction capacity. Starting components for the synthesis of these extractants are often difficult to access [29]. The study was based on a method for extracting copper(II) ions described in [25]. Results of copper extraction are shown in the Table 2.

**Table 2:** Copper extraction results

Samples	Cu content (ppm)					
	Content in the aqueous phase	1st extraction	2nd extraction	3rd extraction	D	%E
		Content in the aqueous phase	Content in the aqueous phase	Content in the aqueous phase		
Cu ore	33802 ± 3281.2	319 ± 25.5	3 ± 0.2	<0.03 below det. limit	107 ± 8.6	99.99 ± 0,01
Flotation tailings	9979 ± 798.3	55 ± 4.4	0.3 ± 0.02	<0.01 below det. limit	179 ± 14.3	99.99 ± 0,01

The analysis of the above mentioned parameters with those extractants allowed for selecting the best conditions. This required three cycles of extraction with three cycles re-extraction of copper to the water phase by 5% Na_2_CO_3_. Results of the distribution ratio shows that, for the separation of copper, the application of a two extraction step is sufficient.

In the case of systems made up of two phases for which the value of the distribution ratio D ≥ 100, a two steps extraction process is sufficient to obtain the transfer of a compound from one phase to another. A value %E is very high approximately >99.9%.

The results show a complete transfer of copper ions into the organic phase, which is equivalent to c.a. 100% recovery of copper from the leaching liquors. Copper in the organic phase can be separated, after re-extraction to the acidic aqueous solution, by electrolysis.

### Extraction of V, Mo, U

**Fig. 2:**
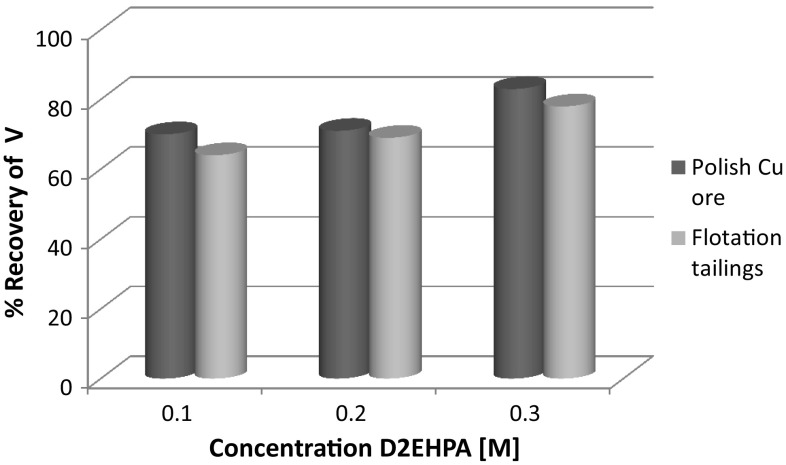
Comparison of vanadium extraction efficiency for D2EHPA, depending on the various concentration of extractants

**Fig. 3:**
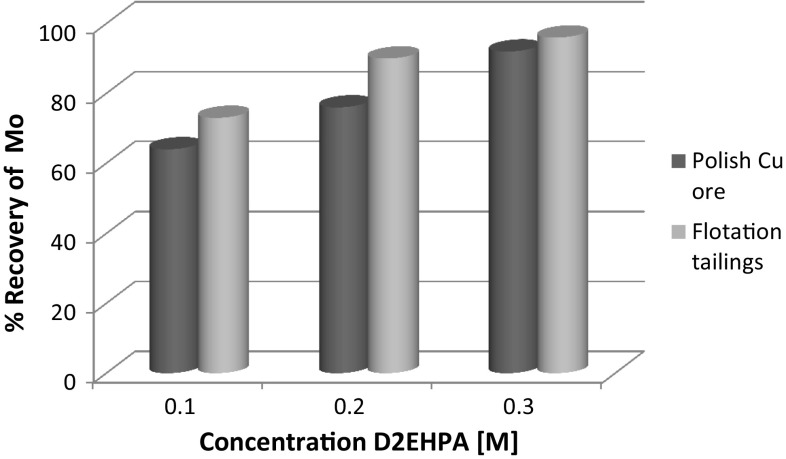
Comparison of molybdenum extraction efficiency for D2EHPA, depending on the various concentration of extractants

**Fig. 4:**
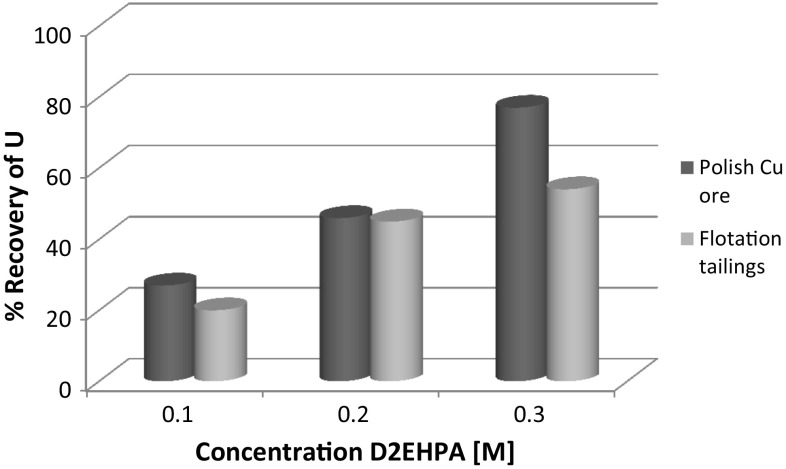
Comparison of uranium extraction efficiency for D2EHPA, depending on the various concentration of extractants

We compared the results of extraction of various metals from samples (leaching of ore and waste with sulfuric acid) for different concentrations of D2EHPA. The copper was recovered from leaching liquor approximately in 100% (Table 2). The results of extraction of vanadium, molybdenum and uranium are shown in Figs. 2, 3 and 4.

Vanadium extraction efficiencies were relatively low, at about 65% for flotation tailings, but in the case of copper ore reached levels of 80%. In the case of molybdenum, recovery was in the range 60–90%, but it was a higher for flotation waste.

Uranium extraction efficiency as a percentage of element concentration in the leach solution varied in the range from 18 up to ~75%. For the solutions with D2EHPA as extractant, the highest recovery rate of metals was observed at the concentration of 0.3 M.

**Fig. 5:**
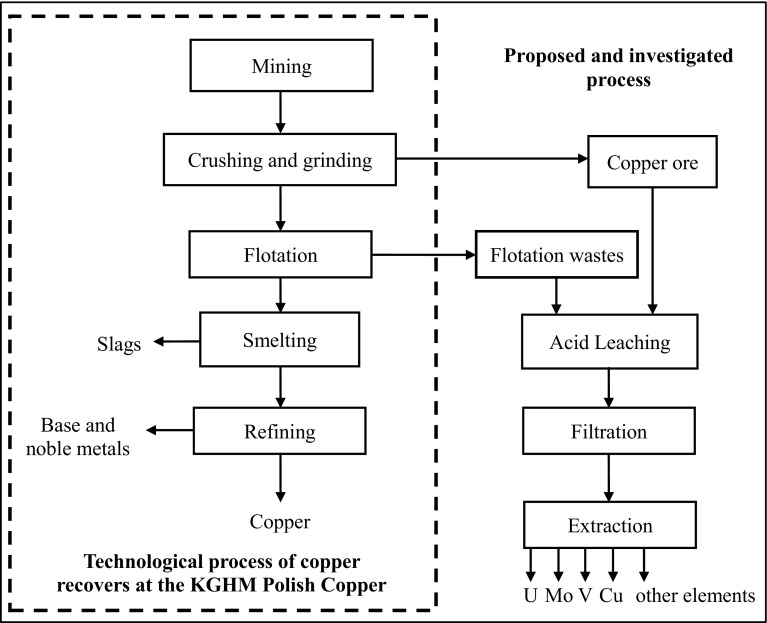
The proposal process for the processing of flotation tailings or Cu ore

Therefore, D increases with an increasing extractant concentration (Table 3). It is then preferable to have a high extractant concentration for better loading of the organic phase with the metal. The integrated process of extraction and re-extraction conducted in continuous mode is now under investigation. In this paper is proposed a hydrometallurgical process that can be a more universal method, which does not require special treatments for various materials such as ore or waste, and may give an additional economic benefit by processing tailings already formed. The proposed solution is illustrated in Fig. 5 with reference to the process of obtaining copper by KGHM.

**Table 3:** Distribution ratio for extraction of V, Mo, U

Distribution ratio				
Concentration D2EHPA	Sample	V	Mo	U
0.1 M	Cu ore	2.33 ± 0.100	1.78 ± 0.072	0.37 ± 0.015
	Flotation tailings	1.78 ± 0.073	2.70 ± 0.116	0.25 ± 0.010
0.2 M	Cu ore	2.45 ± 0.102	3.17 ± 0.123	0.85 ± 0.034
	Flotation tailings	2.23 ± 0.091	9.00 ± 0.378	0.82 ± 0.034
0.3 M	Cu ore	4.88 ± 0.210	11.50 ± 0.495	3.35 ± 0.134
	Flotation tailings	3.54 ± 0.145	24.00 ± 0.040	1.17 ± 0.048

## Summary

The waste tailings from a mine still contain a significant amount of copper. Tenths of a percent of copper contained in tailings stored in waste reservoirs have no value from the point of view of pyrometallurgy. However, these concentrations, relating to millions of tons of waste, currently represent thousands of tons of toxic metals. This is a kind of deposit of copper, molybdenum and other rare metals for future generations. In such cases, as one of the stages of the process of extraction and cleaning of certain elements, hydrometallurgical methods using properly selected, selectively acting extractants are useful. In the present study, we compared the extraction of various metals from solutions of sulfuric acid which had been leached from representative samples of Polish ore and flotation tailings from Gilów.

Extraction tests of elements such as V, Mo and U by D2EHPA were carried out. The results of recovery of these elements in the case of both extractants were satisfactory. Full transition of copper into the organic phase confirmed 100% recovery from leaching liquor. The radiotracer method was a very useful analytical tool that allowed us greatly to accelerate the analytical work. The optimum efficiency of the extraction process was achieved by adjusting optimal parameters. The results can be used as a guide in the design of processes connected with the recovery of Cu and accompanying elements from flotation tailings after the processing of copper ores.
